# Body *v*. Mind

**Published:** 1858-01-01

**Authors:** 


// THE JOURNAL

OF

### PSYCHOLOGICAL MEDICINE

AND

MENTAL PATHOLOGY.

JANUARY 1, 1858.

### Art. I.—BODY *v.* MIND.

It is a curious and interesting study to trace the variety of opinions which have been held concerning the respective existence and the mutual relations of the Body and the Intellectual Principle—opinions which have, in turn, taken up every position between the absolute non-existence of Mind, save as a form or function of Matter, on the one hand; and, on the other, the merely phenomenal existence of Matter dependent upon the variations of a sentient or thinking immaterial existence, the Mind. It was only at a comparatively late period in the world's history that Mind obtained from philosophy its formal recognition as a distinct entity; as something superadded to, and distinct from, Matter; closely united, yet not allied; dependent for its manifestation, but independent in essence. In these latter days, when Mind and Matter are the watchwords equally of domestic discussion, of rival though friendly schools of philosophy, and of fierce sectarian controversy, it is difficult for us to realize to ourselves the state of the schools in which the laws of human nature were taught as a great whole. Yet so it was; it was with man as with the universe at large—he must be one and undivided. As the first attempts at the formation of systems of cosmogony were too vast in their designs to do less than account on one theory for the whole cosmical phenomena, the formation of the universe was ascribed to *one* principle, as heat, atoms, attraction and repulsion, fire, harmony, numbers, &c., no note being taken of the ever progressive workings of the individual forces continually in operation throughout nature, or the mechanical results of the conditional existence of matter.

Such being the case in the macrocosm, we need feel little surprise that the microcosm, man's *superficially* homogeneous nature, should remain long unanalysed; still less, when we consider what an utterly inexplicable phenomenon is involved in its analysis into a material mass, and an immaterial active principle; no less than that something invisible, impalpable, indetectible by any accessible means of investigation, must take possession of a mass of inert matter, and do with it whatever may seem good unto it. Rather may we wonder at the boldness and originality of conception which led Anaxagoras, in the fifth century before the Christian era, to proclaim, in the face of all the incomprehensible theories of the earth and man, that a Supreme Intelligence, or Mind, was the cause of all those phenomena hitherto attributed to Fate, Chance, or some other shadow of a name; and that man was a compound being, consisting of a body and a spirit. It is true that the bubbling, seething, restless, explosive mind must have made itself felt to many, in constantly asserting its supremacy over matter; the difficulty of accounting for these things seems to have been obviated by considering the soul as consisting of finer atoms than the body; and no distinct enunciation of a separate principle was attained to. All honour, then, to Anaxagoras, worthily surnamed by his contemporaries, νους, or Intelligence. Wild and impossible as were his notions of natural causation; eclipsed as was his glory by that of his great pupil and successor, Socrates; yet to him belongs the almost matchless merit of announcing, amidst a heathen world, and without the light of any external revelation, the primitive conception of a One Omnipotent Creative Cause.*

From this time forward, the ψυχη,† or *anima*, which had

* Socrates appears to have been dissatisfied with Anaxagoras, because he could not fully apply his own conception to the practical explanation of nature's mysteries. In the Phaedo, speaking to Cœbes, he says—"Having once heard a person reading from a book written by Anaxagoras, which said that it is Intelligence that sets in order and is the cause of all things, I was delighted with this cause, and it appeared to me to be in a manner well that Intelligence should be the cause of all things." He then proceeds to state how he expected that the author would be able to explain all phenomena according to this intelligence, by considering how it would be best for such and such things to exist, seeing that so they must be best if thus ordered; also, "That he would instruct me whether the earth is round or flat, and would explain the cause and necessity of its being so," &c. "I was in like manner prepared to ask respecting the sun and moon and stars, with respect to their velocities, &c.; in what way it is better for them both to act and be affected as they are." . . . . "From this wonderful hope I was speedily thrown down, when, as I advanced and read over his works, I meet with a man who makes no use of intelligence, nor assigns any cause for the ordering of all things, but makes the causes to consist of air, ether, and water, and many other things equally absurd." In this manner Anaxagoras appears to have been in advance of Socrates, though he could not fully wield his own idea.

† A contemporary writer makes the following remarks illustrative of this subject:—"We do a certain Greek word the honour to translate it *soul;* but it is in fact equally applicable to the vegetative life of a cabbage, to the animal life of a sheep, and to the spiritual life of an apostle. An ordinary Greek thought his body just as much of the essence of his humanity as his spirit, and bodily just as important as spiritual perfection. If St. Paul's thorn in hitherto appeared to be almost equally applicable to man and the brutes, and even to vegetables, had a more specific significance; and man's compound nature became almost imperceptibly a recognised dogma of philosophy.

It is often the case, in the earliest endeavours after truth, that the practical advantages are by no means commensurate with the actual progress made in knowledge. Under the early errors as to man's nature, the body was carefully trained along with the mind; both were treated as fellow-workers in one cause. The Academe, the Lyceum, and the Cynosarges were schools for the body as well as the mind—there the wrestler, the discobolus, and the philosopher met for common purposes.

Under the advanced views, the body became gradually neglected and despised, though this result was naturally of tardy growth. Slowly, however and certainly, the supremacy of mind was acknowledged; a powerful impulse was also given in the same direction by the diffusion of Christianity, and especially by the gorgeous visions of a glorious immortality which were opened to the astonished minds of men awaking from a long Pagan night. Body and mind were thenceforth held, by philosopher and Christian, to have separate and antagonistic interests. To the former, the body was a clog, an impediment to the acquisition of knowledge—a something perpetually interfering, by its pains, its sorrows, and its imperfections, with the clear views of truth which he supposed the unencumbered soul would obtain—constantly distracting the attention by its material relations and requirements—ever of the earth, earthy—tending to its own source, binding and dragging the soul along with it.* To the latter, the Christian, the body was sin incarnate, the source of all evil and temptation, the barrier between the soul and heaven.

Epictetus may well illustrate the views of the philosopher. When severely treated by his master, Epaphroditus, under the most intense agony he smiled, and told him that he would break his leg with twisting it. This actually did occur, but without disturbing his equanimity. On being questioned as to the cause of the flesh was a visible deformity, a Greek educator would have thought it better for him to be put to death as soon as he was born, than to live a burden and a disgrace to his community and to himself. Plato himself would have regarded it as an abuse of the art of medicine to cherish the flickering flame of life in a Pascal or a William III. Epictetus summed up all that was most startling and paradoxical to a Pagan ear when he said, in his own lines on himself—'I was a slave, a *cripple*, a beggar—and a favourite of the gods.' "—*Saturday Review*, Nov. 7, 1857.

* . . . . Noxia corpora tardant,

Terrenique hebetant artus, moribundaque membra.

Hinc metuunt, cupiuntque, dolent, gaudentque, nec auras

Suspiciunt, clausæ tenebris et carcere cæco.

this astonishing composure, he merely replied that the body was "*external*."*

In the early centuries of the Christian era, the body seemed to be ever of less and less estimation. There is something even amusing in the excess of contempt in which it was held, and the abuse heaped upon it. A prison-house, a cage, a weary load of mortality—these were, by comparison, complimentary terms. Gregory Nyssen calls it *οσμῆς ἐργαστήριον* "a fuliginous ill-savoured shop, a prison, an ill-savoured sink," as the words are translated by an old divine. It is "a lump of flesh which mouldereth away and draweth near to corruption whilst we speak of it." St. Augustine defines the two natures thus, "Domine, duo creasti; alterum prope te, alterum prope nihil." At the best, the body was considered a workshop for the soul, *εκ του σωματος τη ψυχη φιλοπονησαι*. The torments of the body were so utterly despised, as scarcely to be considered personal matters:—

Tormenta, carcer, ungulæ,

Stridensque flammis lamina,

Atque ipsa pœnarum ultima,

Mors.

In fine, the body was considered the source of all evil, and, as such, worthy of no consideration. The Platonists, as St. Augustine says, "hold that these our mortal members do produce the effects of fear, desire, joy, and sorrow, in our bodies; from which four perturbations (as Tully calls them) or passions, the whole inundation of man's enormities have their source and spring."†

The Manicheans put the climax to these reproaches cast upon the body. They maintained that the body was so evil that its creation cannot be ascribed to the same author as that of the soul. Farindon says, "The Manichee, observing that war which is betwixt it (the body) and the soul, alloweth it no better maker than the devil;" and Ludovicus Vives, to the same effect, says, "They held all flesh the work of the devil, not of God, and therefore they forbad their hearers to kill any creatures, lest they should offend the Prince of Darkness, whence they said all flesh had originated." In their opinion, the great object of the government of the god of light was to deliver the captive souls of men from their corporeal prisons. But one thing remained to be done after this, and that was reserved for the philosophers of our own era—viz., to deny the body any existence whatever, save as a

* The small estimation in which the body was often held is not obscurely intimated by the question and address of Æneas to his father, who had spoken of souls returning to their bodies:—

O pater, anne aliquas ad cœlum hinc ire putandum est

Sublimes animas, iterumque ad tarda reverti

Corpora? quæ lucis miseris tam dira cupido?

† Translation by Ludovicus Vives.

phase, quality, or affection of the mind. This annihilation, however, it only shared in common with matter in general; in short, with all external nature.

Thus was an antagonism, a division of interests, instituted between the material and the immaterial elements of man's nature—one which, in various forms, in accordance with the spirit of the times, has been propagated even until the present: now one and now the other being held in paramount esteem, in accordance with the demands necessary to be made upon their functions. Here, brain has been had in honour; there, thews and sinews. But the present is essentially an iron and a practical age; both strong limbs and thoughtful minds are in requisition; and the spirit of the age is in nothing more manifest than in the multiform attempts, by the spread of rational education, and the increased attention to the sanitary condition of the masses, to balance the interests of these two hitherto conflicting elements. But according to the infinite varieties of mind, and the different aspects in which these attempts are viewed, there must ever be differences of opinion as to the extent and nature of the remedies applied to existing evils. Notes of alarm are sounded, and responded to; parties are formed; watchwords are in every mouth; discussions, perhaps somewhat acrimonious, take place; finally, out of evil comes good, for the sense of the community is ascertained, and the evil is modified, if not eradicated.

Such is the case at present. Mr. Gladstone, at the conclusion of an address to the members of the Liverpool Collegiate Institution, made some allusion to the antagonism of which we have been speaking:—

"There still remains," he says, "in some quarters a vulgar notion that there is a natural antagonism between corporeal and mental excellence. I trust that corporal education will never be forgotten; that the pursuit of manly sports will always receive the countenance and encouragement, not only of the boys who engage in them, but of the masters who are responsible for the welfare of those boys."

Mr. Gladstone, denying the reality of the antagonism, illustrates his position by the case of General Havelock, who, when at the Charter-house, was one of the quietest of the quiet, "who used to stand looking on whilst others played, and whose general meditative manner procured for him the name of 'Philosopher,' subsequently diminished to 'Old Phlos'"—yet who is now "distinguishing himself by a temper, a courage, an activity, a zeal, a consistency, and a dogged and dauntless resolution, equal at least to that of any man that England has produced this century."

This casual allusion of Mr. Gladstone's gave rise to a powerfully-written but somewhat alarming essay upon the dangers of mental pressure, from our leading journal, which has caused much discussion *pro* and *con*. As this essay embraces the entire case for the prosecution—that is, the whole of the allegations brought by Body against Mind—we shall quote it in great part, as a preliminary to an examination of the question in some detail as to the effect of mental labour upon bodily health, in relation to age, temperament, and other circumstances of perhaps equal importance with either of these :—

"It was a great point in ancient philosophy, the value it attached to the body and the proper training of it, the preservation of its health, strength, and all its proper powers. Ancient philosophy did not despise the body, did not regard it as a mere husk or outside of human nature, or treat it as a despicable and absolutely vile thing; it regarded the body as a true part of human nature, deserving of proper deference, for the failure of which it was sure to retaliate fearfully upon the whole man. Hence the gymnastics of the Greeks, which were not only fostered by the boxers and wrestlers, the drill-sergeants and corporals of that day, but went on under the solemn sanction of sages. There is a distinction between the tone of ancient and modern thought on this subject, and the ancient has certainly an advantage over the modern on this particular point—at least, over the modern before the latest improvements. It has been too much the fashion with us to decry the body, to talk it down, to speak scornfully of it in every possible way, to be always comparing it with the mind for the sole purpose of showing how vile and worthless it is in comparison—a mode of speaking which, even if it is true abstractedly, may be indulged in such a degree as to involve a practical untruth. Our didactic books have been full of the praises of midnight oil, all our oracles of learning have been vehement in favour of unsparing study, and the mind has been subjected to the most acute stimulants, while the body has been left to take care of itself as it can. Of course, the great mass of our school and university youth takes the law into its own hands under these circumstances, and adopts very effective measures against being goaded to suicidal study, but a certain proportion have responded to the whip, and responded but too eagerly.

"These have been the tactics, we say, of our modern masters of the schools and encouragers of learning—an unsparing use of the goad, a merciless appeal to student ambition and emulation, as if it was impossible to stir up these motives too deeply. But how onesided is a discipline which applies this powerful, sharp, and penetrating stimulus to the mind, while it leaves the body to itself, or rather, what is worse, suppresses and flings aside the claims of the body, which has to fare as it can under the exclusive and oppressive dominion of its rival! How partial is such a system, and superficial because partial! After all our sublime abuse of the body, a body man has, and that body is part of himself, and if he is not fair to it, he himself will be the sufferer. The whole man, we say, will be the sufferer—not the corporeal man only, but the intellectual man as well. Particular capacities may receive even a monstrous development by the use of an exclusive stimulus, but the reason and judgment of the man as a whole must be injured if one integral part of him is diseased. If the body is thoroughly out of condition, the mind will suffer; it may show a morbid enlargement of one or other faculty of it, but the directing principle—that which alone can apply any faculty or knowledge to a good purpose, can regulate its use and check its extravagances—is weakened and reduced. How miserable is the spectacle of morbid learning, with its buried hoards, and its voracious, insatiable appetite for acquisition, united with the judgment of a child! Such study does, in short, leave men children with remarkable memories and acquisitive powers, who know as much history, philosophy, and poetry as would make a learned man, but who are not a bit the nearer being men in consequence, because they simply know by rote what they know,—they do not understand their own knowledge. This is to a considerable extent the case with all morbid learning, where the general intelligence has not been cultivated,—which general intelligence depends on the soundness and health of the whole man, body and mind too. The picture of a Kirke White dying at the age of 21 of nocturnal study, wet towels round heated temples, want of sleep, want of exercise, want of air, want of everything which Nature intended for the body, is not only melancholy because it is connected with an early death; it is melancholy also on account of the certain effect which would have followed such a course unchecked if he had lived. We see, when we look down the vista of such a life, an enfeebled and a prostrated man, very fit to be made a lion of, like a clever child, and to be patted on the head by patrons and patronesses of genius, but without the proper intellect and judgment of a man. How sad even is the spectacle of that giant of German learning, Neander, lying his whole length on the floor among his books, absorbing recondite matter till the stupor of repletion comes over him, forgetful of time and place, not knowing where he is, on the earth or in the moon, led like a child by his sister to his lecture-room when the lecture hour came, and led away home again when it is over! Is this humanity, we ask, as Providence designed us to be? Is it legitimate, rational human nature? It can hardly be called so.

"We must not let the mind feed itself by the ruin of the body. The mind has no right to this indulgence, this dissipation, and whole-length abandonment to its cravings, any more than the body has to sensual indulgence. This mental dram, the noxious stimulant which produces this overgrowth of mind, is as contrary to nature as the coarser stimulant which unduly excites the body. The mind should be a good, strong, healthy feeder, but not a glutton. We have no right to despise the body, or to speak of it only and exclusively as something which is vile in comparison with the mind. This language will lead astray. It will make ardent, ambitious student youth neglect health, and abandon themselves to the process of acquisition at the cost of body, and ultimately of mind too. Do not use too unsparingly the motive of ambition in dealing with youth. It is a motive which is perfectly honest and natural within proper limits, but when pushed to excess it produces a feeble, sickly, unmanly growth of character; it creates that whole brood of fantastic theorists, sentimentalists, and speculators which, in art, science, and theology alike, are the seducers and the corruptors of mankind."—*Times*, Oct. 28th, 1857.

The case, though certainly the extreme case, of the injury that *extreme and misdirected* application of the mind may do to the body, is here fairly stated; the illustrations, however, are not fortunate. Kirke White, from his earliest infancy, was of so delicate a constitution as to be unfit (as was supposed) for any active occupation. The question may naturally arise—Would so active and irritable a mind, united to so feeble a frame, have lacked opportunity under any circumstances of rapidly wearing out both itself and its earthly tenement? The wasting fever of such a mind is not to be allayed by any restrictions as to hours of study, rest, or general hygiene. Neander was simply a recluse—a solitary student; nothing worse seems proved or alleged. That he was so absorbed in his favourite pursuits as to be not very conversant with ordinary every-day matters, and even to be a child in many respects, in no respect distinguishes him from thousands of other men whose whole existence is bound up in concerns of much less moment. He lived to a ripe old age, in the enjoyment of moderate health, and all his intellectual faculties, we believe, to the very close of his life.

Setting aside the illustrations, there are some most important allegations, either distinctly expressed or implied, concerning the prominence now given to intellectual pursuits, to the neglect and injury of the bodily health. They amount to this:—

(1.) That mental labour, when approaching to extreme, has an unfavourable influence upon both the health and the character, ruining the former, and rendering the latter "feeble, sickly, and unmanly;" and that this is especially the case with young persons.

(2.) That in our educational systems generally, the body is neglected, and, at its expense, the mind urged beyond its normal powers.

(3.) That in our universities, in particular, the standard of requirements for the obtaining of an honourable or high position is too high.

With the third proposition we are disposed to agree under certain restrictions and limitations; as no doubt many young men, originally of feeble and degenerate constitution, ignorant of any physiological laws, and careless of all hygiene, do break down both in body and mind under the somewhat severe requirements of the curriculum, and a mistaken idea of the true method of mental application with a view to economy. To this subject we shall return shortly.

A writer in the *Saturday Review* has alluded to the second proposition in terms with which we cannot but agree :—

"We are glad that so distinguished an educator as Dr. Kennedy has said a word to allay any undue apprehension that may have been excited as to the neglect of physical development at schools. One would suppose people had never seen the playing-fields of Eton, Harrow, Winchester, or Rugby alive with cricket or football, or the Thames at Windsor on a summer's evening. Those who think that boys at an English public school do not feel respect for distinction in games as well as distinction in Greek and Latin, or that the masters of English public schools do not encourage this feeling, must be ignorant of English schoolboy life. Go to a cricket match at any of the public schools, and look round the ground. You will soon see whether the masters stand aloof from the amusements of the boys—whether to them the physical excellence of their pupils is a matter of indifference or aversion—and whether they grudge every moment which is given to the invigoration of the body and taken from the overstraining of the mind. Some boys there are—as there are some men—who, in spite of all encouragement, and even goading, will not take much part in the sports of their fellows. Sometimes this arises from extreme physical weakness, which may be outgrown in time, but which cannot be cured by force. Sometimes it arises from temperament. Generally it is an unhappy temperament, but occasionally—as in the instance cited by Mr. Gladstone and now before our eyes—it is that temperament of deep thoughtfulness which seems the one indispensable condition of all kinds of greatness. Saving these exceptions—which no system will reduce to uniformity, any more than it will make the colour of all boys' hair the same—we should say the education of English boys at good schools always includes a fair amount of bodily exercise, and that the masters desire and take care that it should do so. Indeed, if we had to name that which in modern times most corresponds to the ancient Greek system of bodily and mental training, we should name the classics and cricket of an English public school."

The first proposition, however, contains the entire pith of the question which is the immediate object of our investigation ; and we propose to inquire what are, from physiological considerations, the probable effects of mental labour upon the bodily health ; what are the actually observed effects; upon what ages, temperaments, &c., these effects are most marked ; what circumstances are calculated to influence for good or for evil the reciprocal actions of mind and body ; and, finally, whether the earnest, or even severe, exercise of the mind may not, both directly and indirectly, be attended by results of a conservative nature, entirely opposed to the views above quoted.

It is scarcely necessary to allude even slightly to the proofs*

* These proofs are in brief derived from the facts, that the brain proper is the one organ which increases from the fish to man in proportion to the intelligence ; that the brain is the material organ (and the only one) through which the mind acts, and communicates with the external world —this is generally acknowledged. It is less understood that the brain, as an organ, is subject to precisely the same laws, chemical, dynamic, and automatic, as other organs and tissues, though physiology teaches this fact as strongly as any other. Thus it is readily granted that the action of a muscle tends to the increase of the circulation in its tissue, and, if long continued, to the hypertrophy or increase of its substance. The same phenomenon takes place in the passive tissues, as the skin, bones, tendons, and ligaments; whenever often-repeated pressure or tension is exercised upon these, their substance is developed in proportion to the requirements of the case.

It is also not disputed that every action of the body is attended by the phenomena of nutrition, including the decomposition of some of the old tissue, and the supply of its place by new particles; and that the evidences of such decomposition in the blood and the excretions are in exact ratio to the energy and continuity of such actions. But although the laws of nutrition are in as active operation in the brain as in any part of the system, we find it at first difficult to realize the fact so well established by irrefragable physiological evidence, that these acts of nutrition are in their essence the necessary conditions of every act of intelligence, perception, or volition; that, "like all other tissues actively concerned in the vital operations, nervous matter is subject to a *waste* or *disintegration*, which bears an exact proportion to the activity of its operations; or, in other words, that every act of the nervous system involves the death and decay of a certain amount of nervous matter, the replacement of which will be requisite in order to maintain the system in a state fit for action;"* in short, that every idea, every emotion, every act of volition, and every perception, however passive or fleeting, is necessarily attended by a waste and decay of a certain portion of the brain tissue. The author just quoted continues thus:—"In the healthy state of the body, when the exertion of the nervous system by day does not exceed that which the repose of the night may compensate, it is maintained in a condition which fits it for moderate constant exercise; but unusual demands upon its powers—whether by the that any part of the nervous system except the brain, or any other organ of the body, may be seriously injured, if not destroyed, and this without any lesion of intelligence; but that all injury to the cerebrum is followed by some lesion of intelligence, perception, or volition. Though the brain alone is capable of manifesting the operations of mind, yet it is not by any means universally held that the "*mental principle*" resides solely in the brain. "It is possible," says Müller, "for the mind to act and receive impressions by means of one organ of determinate structure, and yet be present generally throughout the body."—Vol. ii. p. 817.

* Carpenter.

long-continued and severe exercise of the intellect, by excitement of the emotions, or by the combination of both in that state of *anxiety* which the circumstances of man's condition too frequently induce—produce an unusual waste, which requires for the restoration of its powers a prolonged repose."

It is certainly inexplicable how matter and mind can act and re-act one upon the other; the mystery is acknowledged by all to be insolvable, and will probably ever remain so; the co-ordinate phenomena, however, are open to investigation, and it is clearly ascertained that to certain mental conditions a certain state of the material organ is attached; and for certain mental acts, certain chemical changes in this organ are requisite.

We have stated above, that the brain is subject not only to the same chemical laws of change as the other organs, but to the same automatic influences. In the same manner that certain muscular actions, at first painful, difficult, and complex, become perfectly easy, and are performed almost (if not altogether) without attention after long practice and frequent repetition: so processes of thought, which originally induce painful sensations, and confusion in the mind or brain,* become, by repetition, familiar and simple, and are attended by no pain at the time, nor any inconvenience subsequently. And thus the most complex operations of the mind, calculations involving the most intricate processes, and analyses of the utmost difficulty, are at last performed with an ease, and almost unconsciousness, rivalling the extempore performances of the most finished *artiste* on a musical instrument. It is of importance, in passing, to mark this. We pass on now to notice briefly the various modes in which mind and body affect one another, in order to illustrate the dynamism of the former, and its subjection in many respects to material laws.

A due supply of arterial blood is requisite for the proper action of the mind. Loss of consciousness follows the abstraction of this

* *Mind* or *Brain.*—In a *physiological* point of view, these terms may be used synonymously. The brain is material; the mind is, we conceive, immaterial; yet as we know and can know nothing abstractedly of mind, apart from its manifestations through its material organ, it is convenient occasionally to use these as convertible terms, especially when concerned with laws of action which appear to be connected with, if not dependent upon, material changes. Yet nothing can be more certain than this, that however dependent mind may be for its manifestations upon a material organ, it is essentially different in nature. Were there no presumptive evidence of this from the phenomena of memory, imagination, &c., it would be supplied abundantly by the frequent instances of the persistent integrity of the mind amid the utter decay of the bodily organs. "*My friends,*" said Anquetil, when his approaching end was announced to him by his physicians, "*you behold a man dying, full of life!*" On this expression M. Lordat remarks—"It is indeed an evidence of the duplicity of the dynamism in one and the same individual; a proof of the union of two active causes simultaneously created, hitherto inseparable, and the survivor of which is the biographer of the other."

stimulus. The quality of the blood circulating through the brain also influences the development of ideas—if it be deficient in oxygen, delirium of course follows. "The digestion of food introduces a quantity of imperfectly assimilated material into the circulation; until this new material has undergone the necessary changes, and while certain matters, altogether unfit for nutrition, are mingled with it, it is not adapted to excite those states of the brain which are necessary for the proper manifestation of mind; and as it is conveyed to that organ by the circulation, it produces an injurious change in it, and impedes or disturbs the mental functions. Hence the indisposition to mental labour experienced by some persons after meals."* The same effects are produced, in a more marked degree, by wine, spirituous liquids, narcotics, and the presence of bile or urea in the blood. The organic affections of the brain necessarily and obviously modify the mental conditions, not only by destroying the efficiency of a certain portion of the tissue, but by interfering with the due performance of the organic changes in the other parts.

All this is sufficiently comprehensible, that the organ being deranged, it is no longer capable of performing accurately the behests of the mind. It is much less so, how the derangement of the immaterial essence can affect the organic structure; yet the fact is indisputable. The simplest illustration may be drawn from an occurrence not unfrequent in ordinary experience. A person in perfect health receives a letter containing, perhaps, some fatal news; he drops down, smitten with apoplexy; and after death it is found that the cerebral tissue is torn by an effusion of blood into its substance. Joyous emotion may produce the same or analogous results. A young Frenchman received a complimentary letter from the Directory; he was struck motionless, and his head immediately became affected in a manner from which he never recovered.

The paleness of skin, and weakness of the circulation accompanying the depressing emotions; blushing, and other determinations of blood; excitement of the arterial action, under the influence of anger and the allied passions—all illustrate powerfully and sufficiently the dynamism of mind.

The effect of mental action is forcibly portrayed in Virgil's description of the Pythoness under inspiration:—

"Her colour changed; her face was not the same,
And hollow groans from her deep spirit came.
Her hair stood up; convulsive rage possessed
Her trembling limbs, and heaved her labouring breast.
Greater than human kind she seemed to look,
And with an accent more than mortal spoke;

* Müller.

> Her staring eyes with sparkling fury roll,
> When all the god came rushing on her soul.
> At length her fury fell; her foaming ceased,
> And ebbing in her soul, the god decreased."

Enough has now been adduced to show the powerful influence which states of mind have upon the body; we must now inquire more particularly what are the probable and the observed effects of continued mental labour upon the physical constitution.

In accordance with the physiological principles already enunciated, the first effect of laborious thought will be an increase in the circulation through the brain, and a more active performance of the nutritive functions in that organ, consisting of decay and replacement of particles. Until the brain becomes accustomed to this increased activity of function, it is to be expected that it will be attended with certain unpleasant consequences, as headache and confusion—just as a person who has fatigued one set of muscles by hours of exercise, will feel pain and stiffness in these muscles until, by frequent repetition, the same actions are performed for even a greater length of time with perfect ease, and without any ill consequences resulting. Rest, then, will (it is to be expected on *à priori* considerations) restore the integrity of the cerebral tissue; and frequent repetition of the same mental gymnastics will render easy and pleasurable what was before so difficult and painful. But it may be objected that the brain is a very delicate organ, and much more liable to suffer from over use than the coarser texture of the muscles. This *may* be true; yet it must be remembered that the brain is to be considered as perfectly adapted to the performance of its functions—thought, perception, volition, &c.—as the muscles are to the performance of their varied motions; and if the texture of the one be so much stronger than that of the other, the mechanical injuries to which it is liable are infinitely multiplied.

Pursuing the same expectant reasoning, we shall be prepared to meet with a modification of the tissue of the brain from continued excited nutrition; and owing to the peculiar mechanical conditions of this organ, it can only be, as a general rule, manifested by an increased firmness of texture. Under long-continued application to one class of subjects, we believe the form of the head may be altered even in the adult.* Functionally, we must, of course expect a continually increasing facility and aptitude in all sorts of mental work; whilst from the concentration of the nervous energy upon thought, the tendency to active exertion

* A friend of the writer has had two casts taken of his own head at the interval of several years, during which time he was entirely devoted to artistic pursuits, which he had adopted late in life. The contrast between the two is very striking in the development of certain parts of the forehead and parietal regions.

will naturally become more and more limited. And here we may consider that we meet with the root of the various evils which have been so constantly attributed to mind labour. Not what is *done*, but what is *neglected*, seems to be the *fons et origo malorum.* The weary eye, the cramped limb, the demands of the body—all are neglected, from the all-absorbing nature of the pursuit, and a train of evils must necessarily result, which are naturally enough, but perhaps too readily, laid to the account of mental labour, but which result with equal frequency from all sedentary occupations whatever.

In young persons, the mode of response to stimulus and requirements on the part of the system is somewhat different, both in nature and extent, from that observed in adults. In early life, and up to the margin of manhood, a great part of the energy of the vital functions is devoted to the direct nutrition and consolidation of the bodily organs. The tissues are soft and yielding, and are capable of being very much modified by external agency. If a strain of unusual force be applied, the result is not necessarily, as in the adult, fatigue, which may be readily relieved by rest; but the organ yields, and its efficiency is impaired. Thus the heart over-excited in a child, will become dilated—the bone on which unnatural pressure is exerted, bends—the ligament often or long stretched, yields and becomes relaxed. Now, the brain being subject to precisely the same laws as other organs, as to nutrition, we shall expect to find here also a difference in its response to the calls made upon its action. Long-continued exercise of the mental functions will be attended, as in the adult, by increased circulation and activity of the nutritive functions of the brain; but there is this difference, that the brain tissue here is soft and yielding, and instead of offering the normal resistance to the abnormal afflux of blood, it yields to the pressure, the vessels become enlarged, perhaps permanently, and congestion is the result—productive not only of serious consequences for the time being, but, by the very fact of its occurrence, inducing an ever-increasing liability to its *recurrence.* Then perhaps the overcharged vessels make an attempt to relieve themselves by pouring out some of their fluid contents, and effusion into the ventricles or on the surface of the brain is the consequence. It is easy to conceive, from these considerations, what is the lesson which physiology would teach us in reference to the consequences which may be predicated from intense application in the young. These consequences will be still more marked and serious, if the attention be confined exclusively to one class of ideas; if one faculty be cultivated and urged forward, to the exclusion or neglect of the others.

The testimony of writers on the subject of the effects of mental labour upon the body, is singularly unanimous; none seem to doubt its dire results, especially if commenced young, if pursued long and constantly, and if directed too exclusively to a restricted range of ideas. Dr. George Moore, who has entered deeply into these inquiries, makes the following observations:—

"The brain of a child, however forward, is totally unfit for that intellectual exertion to which many fond parents either force or excite it. Fatal disease is thus frequently induced; and where death does not follow, idiocy, or at least such confusion of faculty ensues, that the moral perception is obscured, and the sensitive child becomes a man of hardened vice, or of insane self-will. . . . . . .

"As the emulative success of classical education is generally dependent on an excessive determination of mind, for the purpose of rapidly loading the memory, it is, of course, attended, for the most part, with a correspondent risk to the nervous system of aspirants after academic honours. Mentally speaking, those who bear the palm in severe universities are often destroyed by the effort necessary to obtain the distinction. Like phosphorescent insects, their brilliance lasts but a little while, and is at its height when on the point of being extinguished for ever. The laurel crown is commonly for the dead, if not corporeally, yet spiritually; and those who attain the highest honours of their Alma Maters, are generally diseased men.* Having reached the object of their aim, by concentrating their energies in one object, an intellectual palsy too often succeeds, and their bodies partake of the trembling feebleness. . . . . . .

"The strongest brain will fail under the continuance of intense thought. All persons who have been accustomed to close study, will remember the utter and indescribable confusion that comes over the mind when the will has wearied the brain. . . . . . .

"The modern system of education appears to be altogether *unchristian;* undoubtedly it contributes much to swell the fearful list of diseases, for it is founded on an unhealthy emulation, which ruins many

* When the mind has been long and actively engaged—if we may use the term, overwrought—a great dislike which is sometimes permanent and invincible may be observed to mental labour of the same nature. We were at a large and celebrated classical school along with several boys distinguished for application, and ranking high in the estimation of an eminent master, by whom they were tasked to the utmost; yet none of them have, to my knowledge, made any figure in life either as scholars or men of business. In the medical profession, we have known students who signally exerted themselves while they were making ready to be examined for a medical degree, but, so far from evincing continued pleasure in scientific pursuits, they have since degenerated into mere traders. In a justly-celebrated university, in which the examination for a Fellowship requires a length and closeness of application which is sufficient to impair the power of most minds, it has been observed that many of the Fellows after their election have lost all their original relish for learning, and have become men of little performance, although originally of great promise.—*Dr. Cheyne, on Partial Derangement of the Mind.*

both in body and in soul, while it qualifies none the better either for business, knowledge, usefulness, or enjoyment, but rather, together with the influence of the money valuation of intellect, causes the most heroic spirits of the age to hang upon public opinion and the state of the market. . . . . .

"We know that determination must vastly excite the brain, when the student or the statesman is induced, by desire for doubtful distinction, to spend his days and nights in the distractions of alternate hopes and fears. Under the strain of these conflicting passions how many a mighty mind sinks into insanity, amidst the mysterious darkness of which some demon whispers close to the ear, 'No hope, no aim, no use in life—the knife is now before you!'"*

These are frightful accusations against study and the present system of education; yet we quote them at length, because they are but the echo and *résumé* of the charges which have been entered against such pursuits, both before and since Festus accused Paul of being mad through much learning. Both amongst ancients and moderns it has been the practice to accuse study, as one of the most frequent causes of madness. Fernelius and Arculanus enumerate "study, contemplation, and continual meditation," as especially tending to mania; "of all men," says Lemnius, "scholars are most subject to it;" and Rhasis adds, "Et illi qui sunt subtilis ingenii, et multæ præmeditationis, de facili incidunt in melancholiam." Origanus says, "contemplatio cerebrum exsiccat et extinguit calorem naturalem, unde cerebrum

* We need make no apology for quoting entire the following passage from the *Scarificator*:—"There is that which destroys more fatally than continued physical exertion. The tendency that over brain work has to destroy the intellect has been long observed. Southey died in darkness from over toil. Walter Scott—he who Anglo-Saxonised the language of Europe, and made a literature—broke down near sixty, and went to his grave with a soft head. 'Tis but the other month a young Scotchman died in London, worn out, his mind a blank from literary toil. And who can doubt it? Angus B. Reach—a clever, witty fellow he was—might have laughed much longer, and made others laugh too, if he had only taken half care of himself!—

> 'From Marlborough's eyes the tears of dotage flow,
> And Swift expires a driveller and a show'—

A soul in ruins—those mysterious, appalling afflictions, laying desolate and waste 'minds that could wander through eternity,' have made us pause and wonder at the awful dispensations of an All-wise Providence, and for a moment doubt their justness. The continued tear and wear, the constant demand for more, more, more, sets the cerebral mass 'on fire.' 'My brain is burning—I can bear life no longer!' said the author of the Old Red Sandstone, and shortly ceased to exist. Strange, some said, how Providence should have allowed such a man to pass away from earth in such a manner; but when we consider the subject philosophically, there is nothing mysterious in it, however much we may regret the circumstance. Providence acts by general, not by special laws. Hugh Miller was, intellectually, a giant, and, physically, possessed a frame of iron; but he violated the laws which govern health—he demanded more work from his brain than it could well perform—it reeled and staggered, but it reeled and staggered in vain. He pulled away, and lashed it into fury, and he perished to gratify his genius and his ambition!"

frigidum et siccum evadit quod est melancholicum. Accedit ad hoc, quod natura in contemplatione, cerebro prorsus cordique intenta, stomachum heparque destituit, unde ex alimentis male coctis, sanguis crassus et niger efficitur, dum nimio otio membrorum superflui vapores non exhalant." In this, spite of its antiquated physiology, there is much sound sense, still indicating in reference to the subject at issue, that it is the omissions, not the commissions, that are the chief sources of evil. Machiavel, however, holds the direct influence of study in weakening the body, dulling the spirit, and abating the strength and courage. Quaint old Burton relates that "a certain Goth, when his countrymen came into Greece, and would have burned all their books, he cried out against it, by no means they should do it, 'leave them that plague, which in time will consume all their vigour and martial spirits.'" Descuret, in his "Medicina delle Passioni," speaks of the results of the study mania (mania dello studio) as loss of memory, epilepsy, catalepsy, madness, sudden and premature death; saying that "lo studio, cibo dell'anima, esige per parte nostra grande sobrietà, se vogliamo che non si trasformi in veleno, la cui azione midiciale non è meno funesta al morale che al fisico." Few writers now venture to speak of study with St. Augustine as "scientia scientiarum, omni melle dulcior, omni pane suavior, omni vino hilarior;" or with another old worthy, "Studia senectutem oblectant, adolescentiam alunt, secundas res ornant, adversis perfugium et solatium præbent, domi delectant," &c., and yet both these had tried it to an extent not often reached in these modern times. To return to modern writers; Müller states as the physiological effect of excessive exercise of the mind, that it "diminishes the activity of the nutritive processes;" we find him remarking however, shortly afterwards, that "the culture of the mind by observation and varied attainments has an ennobling influence on the corporeal frame, and particularly on the lineaments of the face."

M. Tissot brings an enormous list of accusations against the over application of the mind, with many interesting illustrative cases. A young gentleman had given himself up to metaphysical pursuits, which he pursued with ardour, notwithstanding that he felt his health failing. At last he fell into such a condition, that he appeared to see nothing, hear nothing, and spoke not a word for the space of a year. He says that he has seen "very promising children, who have been forced to study so constantly by severe masters, that they have become epileptic during the rest of their lives." On this subject Sir H. Holland says, "In the course of my practice I have seen some striking and melancholy instances of the exhaustion of the youthful mind by this over-exercise of its faculties. In two of them, unattended by any paralytic affection, or other obvious bodily disorder than a certain sluggishness in the natural functions, the torpor of mind approached almost to imbecility. Yet here there had been before acute intellect, with great sensibility; but these qualities forced by emulation into excess of exercise without due intervals of respite, and with habitual deficiency of sleep."

Galen mentions a grammarian who was seized with an epileptic fit when teaching or thinking intently; and Hoffman mentions a young man who had a momentary fit, whenever his mind or his memory was overloaded. Petrarch suffered in a similar manner. The evils charged further by M. Tissot upon intense study are gout, premature baldness and grey hairs, phantasms, delirium, mania; "tumours, aneurisms, inflammations, scirrhosities, ulcers, dropsies, headaches, drowsiness, convulsions, lethargy, apoplexies, and the want of sleep," besides many other secondary results.

But it is time to leave this enumeration of evils, and to inquire with what amount of justice they are attributed to mental work, —under what counts of the indictment mind must plead guilty —whether there are any, and what, extenuating circumstances— to what the verdict of not guilty, or at least not proven must be returned—and whether there may be found some remedy of easy application for the evils which confessedly exist, however caused.

We will commence with an examination into the circumstances connected with education in early life, suppose up to the age of 15. It is not to be denied, and we have already given in our adhesion to the opinion, that intense study in early life is likely to be very hurtful in its consequences; and the practice of forcing and urging the faculties of children into premature development cannot be too strongly and earnestly deprecated. Yet we believe that this practice is by no means so common as has been represented.*

That children are overworked occasionally is notorious; but for this there are other causes in operation besides either force or the principle of emulation. The recent publication of the regulations of the University of Oxford "Concerning the examination of those who are not members of the University," may fairly be supposed to furnish an average standard of the requirements of

* The writer is intimately connected with one large establishment where upwards of two hundred youths from eight to twenty years of age are educated; the inducements to study and to excel are strong and valuable, but the discipline is mild and judicious. During ten years of careful observation, he can recal but three or four instances of any injury resulting from severe mental application: none in which more than a temporary cessation from study has been requisite; and with one exception, all occurring in subjects where there was every reason to suspect a morbidly excitable organization. The exception was that of a naturally very dull boy, with a strong desire to overcome difficulties, which, in fact, were only such to him.

education for boys under fifteen and young men under eighteen; and a perusal of these will at once show that boys of average capacity need exert no extraordinary pressure in preparation. Reading, writing from dictation, parsing, short composition, and the first four rules of arithmetic are the substance of the first five articles: the sixth and seventh comprise a very elementary knowledge of geography, and English history. Eight subjects are then given for selection; the candidate must be examined in one at least, and not in more than four, to be chosen by himself. Latin, Greek, French, and German are the first four subjects, in each of which the exercise is simply the translation and parsing of a passage in one of the most elementary school books, and in all but Greek, the translation of an English passage into the others. The fifth is mathematics, including the first two books of Euclid, arithmetic, and simple equations. Sixth, elementary mechanics. Seventh, chemistry. Eighth, botany and zoology. In all this there is nothing very oppressive, even if required from boys of thirteen rather than fifteen; but of course the curriculum of many of our schools is very much more comprehensive than this; and in them we meet not unfrequently with over-tasked brains, and the consecutive train of evils. But how has this been brought about? Is it necessarily by compulsion, or the goad of emulation? or may there not be a much deeper source of the evil?

Two boys, brothers, enter a large school, are placed on the same form, and are subject to the same regulations, the same tasks, the same inducements. One is studious, cares little for outdoor amusements, and perhaps breaks down in health even before accomplishing the object of his ambition. The other is more given to sport or play than to work; he *may be* a blockhead; but on the other hand he *may* take a respectable or even a high position. The question may fairly be asked—"Whence arose this difference?" Not, clearly, because one was goaded and the other not. Why did the first boy prefer his books to the football or cricket? Plainly because his organization was weaker in stamina than that of the other; exertion of an active character was a toil; the mind *or* the body must be occupied, and as he cannot exert the one, the other must bear the burden. On some occasion, by momentary excitement, he is drawn into some arduous play. Look at him when it is over. He sits down upon a stone, or leans against a wall, his face almost ghastly in its pallor, his hand pressed to his side, his temples throbbing, and gasping for breath. He returns to his books, to which he thenceforth clings as his best friends; yet this mischief is not the *result* of his mental application, both the one and the other are the result of a feeble physical frame, which is now undergoing a process of probation, of which none can predicate the termination.

He may break down, or he may become an intellectual giant; but should the former be the case, study, for which he was apparently better fitted than for anything else, can scarcely be blamed. Would not an active life have been a still shorter one? For such constitutions there is much hope, if they can be placed under intelligent care, and individually watched, guarded, and assisted; but amongst the masses this is as a rule impracticable; there is no resource but the school, where general laws must be in force; and it is a question whether, were the standard of requirements lower, this individual class of mind would be less subject to pressure.

We have nothing to say on the subject of cramming the minds of mere infants with heterogeneous learning. The evils of such a course are utterly incalculable;* but so obvious that those who do not instinctively recognise them, would most probably be impervious to any argument. The stunted and deformed mind and body of the child will presently furnish a reproach bitter enough, and a lesson too late to be practical.

There is one other consideration of extreme importance to be urged in extenuation of the morbid influence supposed to be exerted by early mental culture upon life and character. We have in a previous essay,† pointed out, that in certain portions of all classes of society there are elements of degeneration at work, tending to the extinction of races or families. We are perpetually meeting with the last term of these vanishing series, and witnessing the circumstances attendant upon their final disappearance. Young people in all ranks, with and without education, die daily, the victims of these hereditary influences; certainly, we may affirm, with much greater proportional frequency in those classes where education is, and must necessarily be the exception rather than the rule. When those die whose minds have been left to lie fallow, we attribute their death probably to the real cause; but under circumstances of individual taint precisely similar, when the studious child dies, we ascribe the event to their studies in great measure. It is a note-worthy phenomenon that amongst these degenerate beings, previous to extinction, there is often a remarkable development of certain faculties,

* There are certain exceptional cases on record, proving that extreme precocity is not necessarily and invariably connected with early decay. One such is that of the archæologist Visconti, who died in 1818, ætat. sixty-seven. He knew his alphabet at eighteen months old, and could read Latin and Greek fluently before completing his fourth year. Bentham read Rapin's *England* when three years old, and at eight was a proficient on the violin. He lived to the age of eighty-five. Goethe, Scott, and Franklin, each in early childhood evinced decided indications of the talents for which they were distinguished in after life. Two of them lived to extreme old age, and the third to sixty-two. Many other instances might be adduced, but these are sufficient to illustrate the principle.

† On the Degeneracy of the Race. April, 1857.

amounting to genius. This is alluded to in a passage from M. Morel's work previously noticed:—

"Il existe des individus qui résument dans leur personne les dispositions organiques vicieuses de plusieurs generations antérieurs.

"Un developpement assez remarquable de certaines facultés peut quelquefois donner le change sur l'avenir de ces malades; mais leur existence intellectuelle est circonscrite dans certaines limites qu'ils ne peuvent franchir."

Such cases as these have generally a short and brilliant career; and it is of such that the remark is so frequently made, "What promise of future greatness is here nipped in the bud!" Than this, nothing can be as a rule more mistaken—the fiat of early dissolution is written on the degenerate organism; a lurid phosphorescent light accompanies its decay, a light of which decay is as necessary a condition as is the marsh to the *ignis fatuus;* and if by any means this downward tendency be stopped, it is extremely rare that the autumn of life fulfils the promise of its spring. The life is short, not *because* the intellectual development is precocious or forced, but it is short *and* bright from a common cause deeply engrained in the original exceptional organization.

There are, however, certain unhappy cases where the ambition for intellectual distinction is *directly* concerned in destroying health; these are they, where the ability is not equal to the aspirations, and where the feeling of incompetence leads continually to more and more strenuous exertions. The boy of talents below mediocrity, and with a strong desire (from whatever motive) to excel in certain pursuits, is indeed in a pitiable case,* and will rarely escape serious injury. And this is by no means confined to early life. We know of few more melancholy objects for contemplation than a man—or, as it very frequently happens, too, a woman—inspired with a love for a certain art, poetry, painting, or music, mistaking this love for talent, and wearing out life in hopeless efforts at performance—ever failing, yet sometimes happily unconscious of the failure—trying again and again, yet ever again coming far short of even their own imperfect ideal—finally succumbing, worn out by constant attrition against the rock of the impossible. How many of these bruised and broken spirits will the experience of every thoughtful and observant man suggest to him!

If we now inquire more particularly into the circumstances attendant upon university education, and the charges brought against the severity of the requirements for high honours, we shall

* The case is strictly analogous to that of a weakly or lame boy wishing to excel in running or jumping; a sad instance of which kind of perverted vanity was observed in Byron.

find that very much the same limitations are requisite in our adoption of these views, as in the case of children. Men sink under the course not from the direct influence of mental application, but because they have not the stamina to bear even moderate exercise of the mind—because they are of degenerate constitution, favouring irregular circulation and congestions—because being such, their aspirations are too high for their powers—and because, feeling all this, they are prone to neglect the most ordinary rules of hygiene. The pale, timid student, who labours under continual fear of being plucked, and by night and by day crams his mind with all sorts of miscellaneous knowledge, which it would require a much more powerful intellect to analyse and arrange—he can with no justice be held up as a proof that the requirements of his university are too high.

It is, of course, impossible to say, with any accuracy, what proportion of our youth do break down under the strain on mind and body attendant upon the reading for honours. We are not disposed to deny that many such instances do occur; but still we must maintain, that, not what is *done*, but what is left *undone*, is to blame. The woodman, every now and then, pauses to sharpen his axe—let him neglect this, and continue striking against the unyielding tree with his blunt instrument, and by and bye it breaks. Hear how Ficinus comments upon the thoughtlessness of the bookworm,—

"Solers quilibet artifex instrumenta sua diligentissime curat, penicillos pictor; malleos incudesque faber ferrarius; miles equos, arma venator, auceps aves et canes, cytharam cytharœdus, &c.; soli musarum mystæ tam negligentes sunt, ut instrumentum illud quo mundum universum metiri solent, spiritum scilicet, penitus negligere videantur."

But is not the alarm, on this score, even too great? An able writer, from whom we have already quoted, answers this question, very positively:—

"The mothers and merchants of England need not be in so much alarm for the sanitary condition or the practical character of the promising sons whom they may have committed to the English University system. Reading men at the universities, taken as a class, are so far from being reckless about the state of their bodies, that they are generally very careful of their health. They are more regular than other men in their hours and in their exercise, more abstemious in their diet, more free from vicious habits which injure the constitution. They imitate the candidates for the Olympic wreath in their sobriety and continence, if not in the more active part of their training. We will venture to say nobody would know them from their fellows by their cadaverous appearance. They have among them, as far as our observation extends, at least their fair proportion of men who follow the motto, 'to be ever foremost' in the cricket field, the boat-race, and the tennis-court, as well as in the Senate House or the Schools. So far from being taught by their preceptors to strain their minds to the utmost, and take no care of their bodies, they are constantly warned of the necessity of keeping themselves in good physical order by tutors, private tutors, friends, and all who are interested in their success. Men have the wit to see that good health and spirits are necessary to carry them through the labours of an examination, and that they cannot study to any purpose without a clear head, or secure a clear head without a good digestion and sound sleep. We believe the life of a regular reading man at Oxford or Cambridge, with his eight hours work a day (and no more is needed for high honours), his daily air and exercise, his cheerful society, and his reading party in the Highlands or at the seaside in the long vacation, to be as healthy a life as any—at least as healthy as life in a counting house or a solicitor's office. If there is a little exhaustion immediately after the last examination, three months with a knapsack among the Alps generally sets all right again. The victims of wet towels and strong green tea are, generally, not regular reading men, but gentlemen who have been devoting themselves exclusively to their physical development till within a few weeks of their 'little go,' and are compelled, at last, to put on the steam in preparing for that event. Of course, men are sometimes fools enough to overwork themselves at classics and mathematics, as they are sometimes fools enough to overwork themselves at law or physic; but for one man who has been injured by reading at the University, we think we could point to two who have been injured by boat-racing, and four who have been injured by intemperance, and the other vices to which idleness leads.

"University education is very apt to get the credit of destroying constitutions which, in point of fact, it only finds weak and leaves as it found them. A man who comes up to Oxford or Cambridge with a confirmed and hereditary tendency to consumption, will not be saved, by his Oxford or Cambridge accomplishments, from sinking into an early grave. Nor must a man expect that, by having taken a good class, he will be rendered physically equal to employments to which he and everybody connected with him would otherwise have known that he was physically unequal. A sickly and sensitive youth shows intellectual power, and gets a good place in the class list. Immediately he or his friends take it into their heads that he is to be Lord Chancellor; and he is sent, as Lord Eldon said, to 'live like a hermit and work like a horse,' in order to realize that moderate object of ambition. Being by nature absolutely incapable either of living like a hermit or of working like a horse, he of course breaks down; and then his failure is attributed to University education. If the poet Cowper had been, as he well might have been, a classical first classman at Oxford or Cambridge, instead of being brought up in the most practical way in a lawyer's office, Oxford or Cambridge would have borne the blame of his inability to pass his life cheerfully in lonely chambers in the Temple, and to compete with hard strong natures in the trying arena of the Bar. The fact is, that these men do not lose physical power by being put through a good course of reading—for the simple reason that they never had the physical power to lose. They gain intellectual power, which they might otherwise have never possessed, and are thereby enabled to be at least of some use to the world."

It must not be denied, however, that the tests applied at the present day in our principal universities, to ascertain the attainments of their alumni, are serious matters—so serious that men should have a firm conviction of their strength, before entering so arduous an arena. Strong healthy mind, good "working constitution," temperance in every respect, even in work—all these are essentially requisite. For the brain tissue of a large portion of these workers is still in a condition not so inured and habituated to work that it has become easy, and even second nature—it is still labour.

Perhaps it will not lead us too far from our principal design, to take a survey, as brief as the nature of the subject will permit, of the sort of ordeal through which the candidates for honours at the universities of Cambridge, Oxford, and London, have to pass, chiefly as connected with the B.A.

The first to which we open is the Senate-house examination in January last, at Cambridge—the mathematical tripos. Three hours in the morning of the first day are allowed for the answering of twelve questions, such as the following (No. 8):—

"Prove that in the parabola $SY^2 = SP . SA$.—
A circle is described on the latus rectum as diameter, and a common tangent QP is drawn to it and the parabola; shew that SP, SQ, make equal angles with the latus rectum."

The mathematician will see that although there is nothing very obscure in this, yet the labour of answering twelve such questions in three hours requires a clear head, a ready method, great previous practice, and last though by no means least, a hand of almost lightning velocity, merely to do the mechanical part. It is true that the examiners state, if asked, that *all* the questions need not be answered; that more are asked to give variety, and to afford equal opportunities to different orders of students; yet the aspirant after the highest honours will strive after all; we *know* of instances where all has been accomplished; yet the labour is prodigious.—In the afternoon of the same day, two hours and a half were devoted to twelve other questions; the one following is but the half of No. 10:—

"Prove the formula

$$\text{Cos.}\ (A - B) = \cos. A . \cos. B + \sin. A \sin. B.$$

A being greater than B, and each angle less than 90°."

On the second day the same hours were devoted to twenty-four other questions, of which one must serve as a specimen, it being neither more nor less elaborate than the rest.

"A ray of light passes through a prism in a plane perpendicular to its edge; shew that if $\phi$ and $\psi$ be the angles of incidence and emergence, and $i$ the refracting angle of the prism, the deviation is equal to

$$\phi \pm \psi - i, \text{ or } \psi - \phi - i,$$

according as the incident ray makes an acute angle with the face of the prism towards the thicker end or the edge. Under what convention will these expressions for the deviation be all represented by $\phi + \psi - i$, and with this convention for what value of $\phi$ will $\psi$ change sign?"

On the third day the same, except that in the afternoon there were twenty-two questions, of such a nature and complexity that it appears utterly impossible that the demonstrations to one half of them could even be copied out by the quickest stenographer.

Five more days were similarly occupied, but it would scarcely interest the general reader to follow the course minutely. For those interested in the pursuits and training of our rising graduates, we make only one more quotation, this being part only of one of the last day's questions.

"Assuming the formulæ

$$l\,\alpha + m\,\beta + n\,\gamma = 0$$

$$\frac{l}{\alpha\,(v^2 - a^2)} = \frac{m}{\beta\,(v^2 - b^2)} = \frac{n}{\gamma\,(v^2 - c^2)}$$

investigate the equation of the wave surface in a bi-axial crystal."

Meanwhile the classical tripos requires the translation of Greek and Latin into English and *vice versâ;* the conversion of a passage from Marlowe's "Queen of Carthage" into Greek Iambics, and another into Greek Hexameters; a passage from Cowley, into Latin Hexameters; four verses of the "Hymn to Light," into Latin Lyrics, &c., &c.; and an elaborate series of historical and philological questions.*

These are mental gymnastics of no light order, and he who can come out of the ordeal unscathed and with an honourable position, has shown himself, *ipso facto,* to be *great.* It has been very frequently urged that those men who have attained the highest university rank have rarely been distinguished in after life. We are not prepared to disprove a statement which has been reiterated until it has almost become a recognised dogma; but neither can

* We have given so much space to the Cambridge examination that we have thought it not desirable to enter into any analysis of those of the other two Universities; and in fact there would be but little variety. As is well known, Cambridge is more especially mathematical and Oxford more classical; London is but little behind either in each department. He who could take honourable rank in one, would play a respectable part in either of the others. This is only so far as the general degrees of B.A. and M.A. are concerned. The special degrees, in medicine particularly, require examinations of incomparably greater severity in the London University than in either of the elder sisters. Perhaps there is no more severe test applied anywhere, at least, so far as theoretical knowledge is involved, and practice so far as is possible also.

we receive it as wholly true. Are we mistaken in supposing that Sir R. Peel was almost at the top of the academic tree? And probably many other of our ablest statesmen, could we but refer conveniently to their earlier history. Yet even supposing it to be the case, that the world hears little subsequently of the senior wranglers and the "double first" men, is it necessarily because health and intellect are ruined? Rather may we suppose that the studious and literary habits acquired during years of close application have induced tastes and feelings utterly opposed to the wear and tear of public life; and that the men thus trained prefer rather to occupy themselves with the facts and speculations of nature and philosophy, than to take part in the troublous warfare of politics or polemics.

Are these requirements then, as has been so often said, too severe? Of the most weighty order they are certainly; but we must hesitate before pronouncing them to be too much so. What indeed is a test of this nature intended for, if not to distinguish between man and man? Crowds of men could pass through a lighter ordeal with perhaps equal merit and distinction; and from the nature of things it is inevitable that the severity of the test must be increased till the few can be sifted from the many. Moreover, we must remember that the honour is for those who *can* fulfil the conditions, not for those who *cannot;*—it is for the purpose of selecting the strong and clear-minded man and the one who is capable of much hard work; for such men are wanted in the world, as well as the strong-limbed and hard-handed. All minds cannot accomplish the same feats, no more than all physical frames can rival the material development of a Lydon or a Tetraides. The following is from a recent leader in the *Times,* and well illustrates the various kinds of work and constitution:—

"There is perhaps no man living of whom more facts of labour and triumphs over the frail *physique* of humanity are recorded than of Lord Brougham. Legends of this sort have gathered round him like a Hercules. There is a legend that he once worked six continuous days —*i.e.*, 144 hours without sleep, that he then rushed down to his country lodgings, slept all Saturday night, all Sunday, all Sunday night, and was waked by his valet on Monday morning to resume the responsibilities of life, and commence the work of the next week. A man must, of course, have a superhuman constitution who can do, we will not say this particular feat, which is perhaps mythical, but feats of this class, and probably the greatness of our great men is quite as much a bodily affair as a mental one. Nature has presented them not only with extraordinary minds, but—what has quite as much to do with the matter—with wonderful bodies. What can a man do without a constitution—a working constitution? He is laid on the shelf from the day he is born. For him no munificent destiny reserves the Great

Seal, or the Rolls, or the Chief Justiceship, or the leadership of the House of Commons, the Treasury, or the Admiralty, or the Horse Guards, the Home-Office, or the Colonies. The Church may promote him, for it does not signify to the Church whether a man does his work or not, but the State will have nothing to do with the poor constitutionless wretch. He will not rise higher than a Recordership or a Poor Law Board. 'But,' somebody will ask, 'has that pale, lean man, with a face like parchment, and nothing on his bones, a constitution?' Yes he has—he has a working constitution, and a ten times better one than you, my good friend, with your ruddy face and strong muscular frame. You look, indeed, the very picture of health, but you have, in reality, only a sporting constitution, not a *working* one. You do very well for the open air, and get on tolerably well with fine, healthy exercise, and no strain on your brain. But try close air for a week—try confinement, with heaps of confused papers and books of reference, blue books, law books, or despatches to get through, and therefrom extract liquid and transparent results, and you will find yourself knocked up and fainting, when the pale lean man is—if not 'as fresh as a daisy,' which he never is, being of the perpetual cadaverous type—at least as unaffected as a bit of leather, and not showing the smallest sign of giving way. There are two sorts of good constitutions—good idle constitutions, and good working ones. When Nature makes a great man she presents him with the latter gift. Not that we wish to deprive our great men of their merit. A man must make one or two experiments before he finds out his constitution. A man of spirit and metal makes the experiment, tries himself, and runs the task, as a soldier does on the field. The battle of life and death is often fought as really in chambers or in an office as it is on the field. A soul is required to make use of the body, but a great man must have a body as well as a soul to work with. Charles Buller, Sir William Molesworth, and others, are instances of men whose bodies refused to support their souls, and were therefore obliged to give up the prize when they had just reached it. And how many hundreds or thousands—if one did but know them—perish in an earlier stage, before they have made any way at all, simply because, though they had splendid minds, they had very poor bodies! Let our lean, cadaverous friend, then, when the laurel surmounts his knotty parchment face, thank Heaven for his body, which, he may depend upon it, is almost as great a treasure as his soul. Nature may not have made him a handsome man, but what does that signify? She has made him a strong one."

With this remarkable instance, and many others to which we shall have occasion to refer, before us, we do not hesitate to express again our opinion that the effects of mental application, even of a severe character, are not in themselves so generally serious as it is now the fashion to consider them; and that the greater part of the evils which follow head-work are due to secondary causes, against *some* of which at least it is easy to guard.

The first of these which we shall allude to is the too sudden adoption of extreme studious habits. A man who has for some time neglected his studies, finds himself unprepared as the time of examination approaches; at once he changes all his habits, applying himself the greater part of the day and night to work. Naturally enough, the system rebels against this abuse. The muscular tissue will not bear such treatment; let him try to walk ten or twelve hours in one day without training, and gradually increasing the amount of exercise; and he will be most painfully reminded that organization has its laws which cannot be violated with impunity. The brain tissue cannot be expected to be *more* enduring, or *more* tolerant of such liberties than this; let us but treat it as we would any other organ, then we shall find it as ready to act, and its actions as little hurtful or painful as those. The mind must be *gradually* inured to labour, and then instead of an enfeebled palsied development, we may hope to become able to perform mental athletics to almost any extent without danger, and with ease and profit. It is a most common mistake, in considering the mind as immaterial, to lose sight of this most important fact, that it *acts always* and *exclusively* through the medium of a material tissue; which being, on the one hand, subject to an immaterial essence, does not, on the other, thereby lose its relations to the material organism of which it is an important part.

Another source of evil is the neglect of the corporeal requirements for a great number of hours consecutively. It is almost certain* that the same amount of work which often proves injurious by its continuity, might be achieved with ease, if it were divided by *short* intervals of rest and refreshment. We appeal to the experience of all students, if during their earlier efforts nature did not give broad hints of requiring repose and restoratives;—the stomach asserts its right to food at proper intervals, but it is put off—" go thy way for this time; when I have a convenient season . . . . ;" then when the exhausted powers refuse any longer to work without fuel, the meal is but a business to be accomplished as speedily as possible; the food is swallowed unmasticated, and the stomach, loaded perhaps with a mass of indigestible material, is further impeded in its operations by the immediate resumption of a cramped, constrained, and compressed attitude. Indigestion with its thousand sons is the natural result. Then the head aches, and its hint is evaded by a wet towel, and perhaps an irritating stimulant, as a cup of strong tea or coffee; under the influence of which, temporary power, or a semblance of it, is regained. The weary eye, the aching limb, the general

* The writer would say to the earnest inquirer how much work he can get out of his brain, and how he can do it most safely, that these hints are not drawn from theory. *Crede experto!*

febrile condition—all these are disregarded; day by day the same process is repeated; until the wonder is, not that the brain gives way at length, but that it has held out so long—longer, we venture to say, as an ordinary rule, than any other organ would have done under an equivalent amount of ill-treatment. Yet in all this, the fact of mental labour simply is not more to be blamed than is commerce for the great number of deaths brought about by the all-absorbing desire of gain, the *auri sacra fames* which operates in precisely the same *secondary* manner upon the health and character.

The neglect of fresh air, regular exercise, and early rising, enters into the same category of the secondary causes.

Yet there are other conditions attendant too often upon a literary life, which are inherent in our nature, and in the existing order of our social arrangements, which exert a most important and gloomy influence upon the reaction of mind upon the body; such are the co-operation of poverty, of wearing anxiety, of the depressing passions and emotions generally; and finally, in an overwhelming majority of cases, the pre-existence of elements of degeneracy and disease in the organism.

"Poverty," says old Burton, "is the muse's patrimony; and as that poetical divinity teacheth us, when Jupiter's daughters were each of them married to the gods, the muses alone were left solitary, Helicon forsaken of all suitors, and I believe it was because they had no portion."

> Calliope longum cœlebs cur vixit in ævum?

> Nempe nihil dotis, quod numeraret, erat.

Literature is a "good staff but a bad crutch,"—fascinating, cheering, and enlivening, tending to promote life, health, and an equable mind in those who pursue it for pleasure; but woe to those who are dependent upon their brains for daily bread—thrice woe, if others are dependent upon them. In straitened circumstances, which preclude the possibility of obtaining almost even the necessaries of life—these only to be got by unremitting toil—under the stern necessity for doing so much brain-work in so many hours—for coining, in short, so much nerve tissue into so much, or rather so little, money—pale faces around him asking for bread and shoes—a partner of his woes vainly trying to conceal that she has not wherewithal to procure the day's dinner—who can wonder that, under privation and misery such as this, the powers fail?—who can wonder, or who can venture to blame him, if he sometimes looks forward to the coming of the "Pale Phantom" with something of hope?—who dare but veil his face and pity him if he in some dark moment courts his coming? And when, having to the end kept his faith in his Maker's justice, and fought his good fight, he hears a voice saying "Well done, good and faithful servant," shall we then wonder that he can willingly leave wife and child, to be at rest?

The presence of the seeds of disease and degeneration in the system has already been noticed as a fruitful source of the deaths that so often occur apparently under the influence of studious habits. If these co-operate with the last-mentioned class of influences, the lethal effects will be much more rapid: then early death, or a life of wretchedness often terminated by suicide, is an almost necessary result.

These are sad but apparently inevitable consequences of the conditions of society and of our race. There is, and ever will be, a loud demand for intellect and its labours,*—there exist, and ever will, poverty, and wretchedness, and disease;—in the exhaustless combinations of society these will at times become associated; doubtless for wise and benevolent purposes these things are appointed as amongst our probation experiences; it is not our province to attempt here to "vindicate the ways of God with men;" and an investigation into the proximate causes

* We again quote from the thoughtful writer in the *Saturday Review* of November 7th:—"The ascendancy of mind over physical strength is civilization. Everybody knows that Thersites would now bring down Achilles half a mile off with an Enfield rifle. We need not quote Macaulay's remarks—as brilliant as his remarks usually are, and more true—about 'the hunchback dwarf who urged forward the fiery onset of France, and the asthmatic skeleton who covered the slow retreat of England' at the battle of Landen. Read the chivalrous and romantic Froissart's account of the deliverance of France from the English invaders—you will see nothing but the hand of Bertrand du Guesclin.. Read the true history of the time, and you will see that the real spring of all was the head of that feeble invalid who conquered the two Edwards, to their great amazement, without ever mounting a horse or drawing a sword. It was the dawn, yet unperceived by the Troubadour, of the triumph of intellect over men-at-arms. And power having passed from the body to the mind, ambition itself (to say nothing of higher motives) will mainly cultivate that which is now the real source of power. The development of physical strength will be comparatively neglected, and the body, in this sense, will be sacrificed to the mind. Our material part still asserts its claims, as all who have tried to work with the brain under great physical suffering or debility must know too well; but they are the claims of a servant, not of an equal. Nay, even those gifts of mind which are most akin to and most dependent on bodily health, have a tendency to fall under the dominion of others which are of a more eccentric, and, as a man of business might think, of a morbid kind. You naturally picture to yourself the ideal of humanity—the great man—as a noble bodily presence, full of health and vigour, with a mind as healthy and vigorous as its abode, with all the faculties and acquirements equally balanced, and the soundest judgment sitting supreme over the whole. Look at the records of history and see how far this ideal is fulfilled by the men who have really moved the world. Consider the strange and unsightly caskets in which the rarest and most potent essences of nature have been enclosed. 'Is this humanity,' the practical writer in the *Times* would say of Socrates in his day-long trance of thought, or the macerated and visionary Luther in his Augustinian cell. No, strictly speaking, it is not humanity. It is the upward aspiration of a being of whom mere humanity is the lower and grosser part. It is, in one sense, a sort of disease. But to cure that disease would be to reduce mankind to a mass of money-getting clay."

of these evils would lead us into the fathomless abyss of an inquiry into the origin of evil. We turn to more practical points.

As there are conditions of depression and deterioration in the system which preclude the possibility of long-continued mental labour with impunity, there is, on the other hand, a hardy, vigorous, excited state of rude health, which, so long as it lasts, is as great a barrier to successful hard work. It is not long since we saw a hard-working student, of good sound constitution, who had taken the relaxation of a Continental tour for a few weeks, and who complained on his return that he could not work—his body was too vigorous. Again, the overworked body reacts as powerfully upon the mind, as the overstrained mind does upon the *physique*; hence the toils and anxieties of an arduous professional life too frequently incapacitate the man of moderate powers for any striking intellectual efforts.

It is necessary and useful to inquire what classes of temperament are the best fitted for mental labour, and the most likely to produce satisfactory results. We say, without much hesitation, the Phlegmatic and the Choleric.

Müller, who takes a mental and metaphysical rather than a corporeal view of the various temperaments,* describes the Phlegmatic as one whose "mental strivings or emotions are neither intense nor enduring."—"In persons of this temperament, ideas are conceived with as much rapidity as in others, and there may be the same powers of mind as in other temperaments. When the intellectual faculties are good, this temperament will render a person capable of more difficult acts, and successful in a more extraordinary degree, than were his impulses rendered stronger by a more passionate temperament" (*e.g.*, the sanguine or melancholic). "Such a person, whose mental strivings or emotions are not violent, remains cool and undisturbed, and is not drawn away from his determined course to the performance of acts which he would regret on the morrow;—he is more sure and trustworthy than persons of an opposite temperament, and his success more to be depended on: in times of danger, and at moments of importance, when good judgment, calculation, and reflection, rather than quick action, are needed, his powers are all at his command. When rapid action is required, the phlegmatic person is less successful, and others leave him behind; but when no haste is

* "According to my view, the temperaments are entirely dependent on the different degrees in which different individuals are disposed to the strivings and emotions arising from the depression or excitement of the feeling of self; in other words, in the different degrees of disposition to the states of desire, pleasure, and pain, and on the extent to which these states of the mind are promoted by the composition and states of the organs of the body."—Müller's "Physiology," translated by Baly.

necessary, and delay is admissible, he quietly attains his end, while others have committed error upon error, and have been diverted from their course by their passions."

In the Melancholic and the Sanguine, the chief tendencies of the mind are to the feeling of pain in the former and pleasure in the latter. The Melancholic person suffers impediments to depress and dishearten him, and a corresponding effect is produced on the physical frame. The Sanguine is quick to conceive, but not stable enough for execution ; full of purpose, but fickle and volatile in performance. The system is more formed for activity than for study.

The Choleric has not the indifference of the Phlegmatic, but compensates for this want by the intensity and durability with which he can act. His powers of reflection are less, but his action is prompt, decided, and unhesitating. He has a powerful will, and not given to failure where his mind is once fixed on success. Under the influence of "ambition, jealousy, revenge, or love of rule," his powers seem to have no limits.

The nearer is the temperament, then, to the sanguine or the melancholic, the more care will be required in the adoption of intensely studious habits ; whilst the choleric and the phlegmatic person may with comparative safety, and with ordinary regard to the rules of hygiene, follow the bent of his inclinations—the one, because his constitution is specially adapted to quiet and sedentary pursuits; the other, because his will is sufficiently powerful to govern the functions.

But it is time to inquire whether a negative defence of mind is all that can be brought forward, or whether there are not positive advantages and conservative influences attendant upon mental labour which tend to ameliorate the evils of temperament and constitution, and to prolong life. It is a matter of daily experience, how powerful is the influence of mental application in relieving bodily pain ; how pre-eminently successful it is in soothing the ruffled, troubled spirit, and in softening the asperity of corroding anxiety and care. If the student be poor, his books are his riches ; and whilst living and communing with sages and philosophers, he has no troubles about the state of the funds or the rates of discount. If he be rich, his studies are an omnipotent resource against *ennui*, and will (if aught can do so) prevent that burning desire for *more* which riches so often bring with them.

But mental occupation has a more direct and specific influence upon certain hereditary maladies, of which we may adduce some instances. Burton, himself addicted to the disease, in his "Anatomy of Melancholy," strongly recommends study as a remedy ; and by the catalogue which he gives of things to be inquired into, he evidently does not consider that a man need limit himself. Finding his health and mind failing, he took to writing this work—a perfect miracle of learning, and doubtless by its assistance he lived to the age of sixty-four. Poor Cowper's melancholy was greatly relieved for a considerable time by the writing of the "Task." With the inherent vices of his constitution, and his tendency to the worst form of hypochondria, it is very doubtful whether, without mental labour, sometimes of a severe and almost compulsory character, he would have lived to the verge of seventy. Byron found a necessity for writing to preserve the integrity of his mind. "I must write to empty my mind, or I shall go mad." Accumulated instances would add nothing to the force of the argument; but no one who has suffered in mind or body, and has had resolution to try severe study as a remedy, will doubt its efficiency.*

Let us now inquire what testimony history bears to the longevity of men whose lives have been essentially intellectual. Some objections may be made to this course of investigation; thus we can only quote the most remarkable instances;—we cannot in many cases say how much of the life was purely *studious*—we cannot, in our limits, review the labours of these men—we cannot enumerate those who died young, nor still less can we estimate how many, who would otherwise have been great as these, have failed in physical strength. With all these limitations, we may still hope, by a cursory glance at names which have marked epochs in philosophy and literature, to arrive at some idea of the influence of life devoted to thought rather than to action; and also to prove, by positive instances, that there is nothing in the most intense application which must *necessarily* tend to shorten life, seeing that many of the most laborious men have been octo- and nono-genarians, and even centenarians.

M. Tissot states that Gorgias, the rhetorician, lived to the age of one hundred and eight years, "without discontinuing his studies, and without any infirmity." Isocrates wrote his "Pan-Athenæai" when he was ninety-four, and lived to ninety-eight. The above writer also mentions the case of "one of the greatest physicians in Europe, who, although he had studied very hard all his lifetime, and is now almost seventy, wrote me word not long since that he still studied generally fourteen hours every day, and yet enjoyed the most perfect health."

Epimenides, the seventh of the "wise men," lived, it is supposed, to the age of one hundred and fifty-four. Herodicus, a very distinguished physician and philosopher, the master of Hippocrates,

* "It may be truly said, without any hyperbole, that every pursuit which ennobles the mind has a tendency to invigorate the body, and by its tranquillizing influence to add to the duration of life."—Madden's "Infirmities of Genius," vol. i. p. 112.

lived to the age of one hundred. Hippocrates himself, whose genuine writings alone would be sufficient to testify to a life of arduous study, lived to the age of ninety-nine. Galen wrote, it is said, three hundred volumes; what now remain of his works occupy, in the edition of 1538, five folio volumes. He lived to near one hundred years. Lewis Cornaro wrote seven or eight hours daily for a considerable period of his life, and lived to the age of one hundred, in spite of a feeble constitution originally.

Theophrastus wrote two hundred distinct treatises, and lived to the age of one hundred and seven. Zeno, the founder of the Stoic school, lived to the age of ninety-eight; and, in the full possession of his faculties, then committed suicide, having received, as he supposed, a warning by a wound of the thumb that it was time for him to depart. Democritus was so devoted to study and meditation that he put out his eyes, it is said, that external objects might not distract his attention. He died aged one hundred and nine years. Sophocles died aged ninety-one. Xenophon, Diogenes, and Carneades, each lived to the age of ninety. Varro wrote five hundred volumes, and lived to eighty-eight years. Euripides died aged eighty-five; Polybius, eighty-one; Juvenal, above eighty; Pythagoras, eighty; Quintillian, eighty. Chrysippus died of laughter, at eighty. The poet Pindar died aged eighty; Plato, aged eighty-one. Socrates, in the full possession of his faculties, was judicially murdered at seventy-one. Anaxagoras, to whom we have before alluded, died at seventy-two. Aristotle died at sixty-three. Thucydides was eighty.

It would be difficult to select twenty-five names which exerted a much greater influence upon literature, philosophy, and history, than these in old times. Many of them are known to have been most voluminous writers—many of them most profound thinkers. These were not the days of handbooks and vade-mecums; those who wanted information or mental cultivation had to work for it. Yet the average age of these twenty-five men is exactly ninety years. It is much to be questioned whether the united ages of twenty-five of the most distinguished farmers that the world has ever produced would amount to two thousand two hundred and fifty years. The list might easily be enlarged greatly by such men as Seneca and Pliny, who came to untimely death by accident or tyranny, and who promised to live as long as the oldest, in the course of nature.

We cannot refrain from quoting some remarks upon the labours of the old commentators, which appeared in an amusing paper in a contemporary journal,* before passing in review the ages of some of the most distinguished:—

* Chambers's Journal, Oct., 1857.

"Homer says that it would take nine men of his degenerate day to lift a stone thrown by a single warrior of the heroic ages. We know not how many men of our own time it would take to equal the labour of our commentator—certainly not less than a dozen. In truth, his were the heroic days of literature. See how the pile of manuscript grows under his indefatigable fingers! If he has sat at work less than sixteen hours in the twenty-four, he considers, like Titus, that he has lost a day. 'Fits!' says Bernard Lintot, in Pope's squib against Dennis—'a man may well have fits and swollen legs who sits writing fourteen hours a day.' Alas! the degenerate days had already set in; in the time of Bernard Lintot, our commentator sat writing for sixteen hours, for six months in succession, without having fits or swollen legs. There was a time when he only allowed himself one night's rest out of three. He was warm with youth in those days, and found that he had gone too far; there are stones too heavy even for Homeric heroes. No wonder that piles of folios grew out of his labours."

Yet these old writers, commentators and others, were apparently a hardy race—they were generally long-lived. Beza, the severity of whose enormous labours might be supposed to be aggravated as to the results by the acrimonious controversies in which he was engaged, lived in the perfect enjoyment of his faculties up to the age of eighty-six. The learned Richard Bentley died at eighty-one; Neander was seventy-eight; Scaliger, sixty-nine; Heyne, eighty-four; Parr, eighty; Pighius, eighty-four; Vossius, seventy-three; Hobbes, ninety-one, at death.

Mr. Madden, the able author of the "Infirmities of Genius," has constructed some most instructive tables relative to the longevity of men distinguished for their intellectual pursuits. He says that each list contains twenty names, in which "no other attention has been given to the selection than that which eminence suggested, without any regard to the ages of those who presented themselves to notice."

An analysis of the tables gives the following averages of life for the various classes :—

| | Aggregate years. | Average. |
|---|---|---|
| Twenty natural philosophers . . . . . . | 1504 ... | 75 |
| Twenty moral philosophers . . . . . . | 1417 ... | 70 |
| Twenty sculptors and painters . . . . | 1412 ... | 70 |
| Twenty authors on law, &c. . . . . . . | 1394 ... | 69 |
| Twenty medical authors . . . . . . . | 1368 ... | 68 |
| Twenty authors on revealed religion . . | 1350 ... | 67 |
| Twenty philologists . . . . . . . . | 1323 ... | 66 |
| Twenty musical composers . . . . . | 1284 ... | 64 |
| Twenty novelists and miscellaneous authors | 1257 ... | 62½ |
| Twenty dramatists . . . . . . . . . | 1249 ... | 62 |
| Twenty authors on natural religion . . | 1245 ... | 62 |
| Twenty poets . . . . . . . . . . . | 1144 ... | 57 |

This list does not by any means give too high an average of life for literary characters. Many of the oldest are omitted from the calculations, because, though equally laborious, their eminence was not quite so great; and, again, many are inserted, because eminent, who died young, obviously not from causes connected with mental application. This is particularly illustrated amongst the poets by the cases of Byron and Burns, whose deaths certainly were not justly to be attributed to the nature of their mental habits. Amongst artists, also, Fuseli (eighty-four), Nollekens (eighty-six), Kneller (seventy-five), and Albert Durer (eighty-seven), are not mentioned. M. Lordat, in his "Mental Dynamics," gives many remarkable instances of intellectual pursuits being carried on to an extremely advanced age—"for instance, M. des Quersonnières, one hundred and sixteen years of age, now residing in Paris, an accomplished poet, remarkable for his powers of conversation, and full of vivacity." He mentions also another poet, M. Leroy, aged one hundred years. Fontenelle, considered the most universal genius that Europe has produced, for forty-two years Secretary to the Academy of Sciences in Paris, lived with unimpaired faculties to the age of one hundred years. Father Sirmond, called by Naudé "an inexhaustible treasury of ecclesiastical lore," lived to the age of ninety-three. Hutton, the learned geologist and cosmogonist, died at ninety-two.

We will now give a table of distinguished men, with their ages, independent of classification or chronology—such names as are sufficiently known to the world to preclude the necessity of giving any account of their labours:—

| | Age. | | Age. |
|---|---|---|---|
| Bacon (Roger) | 78 | Hoffman | 83 |
| Buffon | 81 | Pinel | 84 |
| Copernicus | 70 | Claude | 82 |
| Galileo | 78 | Titian | 96 |
| Lowenhoeck | 91 | Franklin | 85 |
| Newton | 84 | Halley | 86 |
| Whiston | 95 | Herschel | 84 |
| Young | 84 | La Place | 77 |
| Ferguson (Adam) | 92 | Linnæus | 72 |
| Kant | 80 | Metastasio | 84 |
| Reid (T.) | 86 | Milton | 66 |
| Goethe | 82 | Bacon (Lord) | 65 |
| Crebillon | 89 | Hobbes | 91 |
| Goldoni | 85 | Locke | 72 |
| Bentham | 85 | Stewart (D.) | 75 |
| Mansfield | 88 | Voltaire | 84 |
| Le Sage | 80 | Cumberland | 80 |
| Wesley (John) | 88 | Southern (Thomas) | 86 |

| | Age. | | Age. |
|---|---|---|---|
| Coke (Lord) | 85 | Rollin | 80 |
| Wilmot | 83 | Waller | 82 |
| Rabelais | 70 | Chalmers | 83 |
| Harvey | 81 | South (Dr.) | 83 |
| Heberden | 92 | Johnson (Dr.) | 75 |
| Michael Angelo | 96 | Cherubini | 82 |
| Handel | 75 | Fleury (Cardinal) | 90 |
| Haydn | 77 | Anquetil | 84 |
| Ruysch | 93 | Swift | 78 |
| Winslow | 91 | Watts (Dr.) | 74 |
| Morgagni | 89 | Watt (Jas.) | 83 |
| Cardan | 76 | Erasmus | 69 |

This list is taken entirely at random, and might be almost indefinitely enlarged; but we are warned to conclude.

There are certain practical deductions obviously to be drawn from the details and arguments that have been brought forward.

1. Devotion to intellectual pursuits and to studies, even of the most severe* and unremitting character, is not incompatible with extreme longevity, terminated by a serene and unclouded sunset. When Fontenelle's brilliant career terminated, and he was asked if he felt pain, he replied, "I only feel a difficulty of existing."

2. Mental application is a powerful remedy in diseases both of body and mind; and its power as a remedy is proportionate to its intensity as a pursuit.

3. The emotions, especially those of a depressing kind, as anxiety, fear, &c., have a remarkable influence in giving a tone to, and intensifying the morbific effects of, excessive mental labour. Yet in some cases, as in those of Byron and Cowper, the best and only resource against despair is found in composition.

4. The turmoils of active life do not appear to render intellectual labour more injurious to the system; possibly here also the influence may be counteracting. Milton, the Secretary to the Commonwealth, in times when men lived years in months—blind and in domestic discomfort, writing his immortal poems; John Wesley, persecuted and almost an outcast from his former friends—in "labours more abundant"—denying himself natural rest and refreshment, yet acting with mind and body with unparalleled energy; Voltaire, the apostle of infidelity, at war with more than the whole world; Luther, hunted by principalities and powers like a wild beast—these and a cloud of others warred with the existing order of things, and remained masters of themselves and their mental powers to a ripe old age.

* Dr. Johnson composed his "Dictionary" in seven years! And during that time he wrote also the Prologue to the opening of Drury Lane Theatre; the "Vanity of Human Wishes;" the tragedy of "Irene;" and the "Rambler"—an almost incomprehensible effort of mind. He lived to the age of seventy-five.

5. The injurious effects of mental labour are in great measure owing—

To excessive forcing in early youth;

To sudden or misdirected study;

To the co-operation of depressing emotions or passions;

To the neglect of the ordinary rules of hygiene;

To the neglect of the hints of the body; or

To the presence of the seeds of disease, degeneration, and decay in the system.

6. The man of healthy phlegmatic or choleric temperament is less likely to be injured by application than one of the sanguine or melancholic type; yet these latter, with allowance for the original constitution, may be capable of vast efforts.

7. The extended and deep culture of the mind exerts a directly conservative influence upon the body.

Fellow-labourer! one word to you before we conclude. Fear not to do manfully the work for which your gifts qualify you; but do it as one who must give an account both of soul* and body. Work, and work hard, whilst it is day; but the night cometh soon enough—do not hasten it. Use your faculties, use them to the utmost, but do not abuse them—make not the mortal do the work of the immortal. The body has its claims, —it is a good servant; treat it well, and it will do your work; it knows its own business; do not attempt to teach or to force it;

* That a mental endowment should retain its vigour, it is necessary that it be moderately exercised. If the exercise of the religious sentiments be interrupted, for example, by too exclusive an attention to science, communion with God will lose its relish. Claudius Buchanan, while at Cambridge, wrote to a friend as follows:—"I find this great attention to study has made me exceedingly languid in my devotional duties. I feel not that delight in reading the Bible, nor that pleasure in Divine things, which formerly animated me. On this account have many serious students in this University wholly abandoned the study of mathematics; for it seems they generally feel the same effects that I do."—*Dr. Cheyne, on Partial Derangement of Mind in supposed connexion with Religion*, pp. 57—59.

It is a great mistake to suppose that men who have obtained great distinction and high honours at our two English Universities, do not in after life occupy the most eminent positions at the bar, on the bench, and in the Senate. First, as to

OXFORD.—Earl of Eldon, English Prize Essay, 1771; Lord Tenterden, (Lord Chief Justice of the King's Bench,) English Essay, 1786, Latin verse, 1784; Sir W. E. Taunton, (Judge in Court of King's Bench,) English Essay, 1793; J. Phillimore, (Professor of Civil Law,) English Essay, 1798; Sir C. E. Gray, (Chief Justice of Bengal,) English Essay, 1808; Sir J. T. Coleridge, (Judge in Court of Queen's Bench,) English Essay, 1813, Latin verse, 1810, Latin Essay, 1813, 1st class Classics, 1812; Herman Merivale, (Professor of Political Economy,) English Essay, 1830, 1st class Classics, 1827; Roundell Palmer, (Deputy Steward of the University,) Latin Essay, 1835, Latin verse, 1831, English verse, 1832, 1st class Classics, 1834; Lord Colchester, Latin verse, 1777; Sir J. Richardson, (Judge in Common Pleas,) Latin verse, 1792; Sir Charles Puller, (Chief Justice at Calcutta,) Latin verse, 1794; G. K. Rickards, (Professor of Political Economy,) English verse, 1830, 2nd class Classics, 1833; Senior Nassault, (Professor of Political Economy,) 1st class Classics, 1811; Sir Richard Bethell, (Attorney-General, University Counsel,) 1st class on the Classics, 1818; Honourable J. C. Talbot, (Deputy High attend to its wants and requirements, listen kindly and patiently to its hints, occasionally forestall its necessities by a little indulgence, and your consideration will be repaid with interest. But task it and pine it and suffocate it, make it a slave instead of a servant; it may not complain much, but, like the weary camel in the desert, it will lay it down and die.

Steward,) 1st double Classics, 1825; Travers Twiss, (Regius Professor of Civil Law,) 2nd double Classics, 1830.

Cambridge.—Sir F. Maseres, (Baron, Exchequer,) 4th Wrangler, 1752, Senior Medallist; Sir Elijah Impey, (Chief Justice, Fort William, Bengal,) 2nd Senior Optime, 1756, Junior Medallist; Sir J. Wilson, (Judge, Common Pleas,) Senior Wrangler, 1761; Lord Alvanley, (Chief Justice, Common Pleas,) 12th Wrangler, 1766; the late Lord Ellenborough, (Chief Justice, King's Bench,) 3rd Wrangler, 1771, Senior Medallist; Sir S. Lawrence, (Judge, Common Pleas,) 7th Wrangler, 1771; Sir H. Russell, (Judge in India,) 4th Senior Optime, 1772; the late Lord Manners, (Chancellor of Ireland,) 5th Wrangler, 1777; Chief Justice Warren, of Chester, 9th Wrangler, 1785; the late John Bell, Senior Wrangler, 1786, Senior Smith's Prizeman; Sir J. Littledale, (Judge in Court of Queen's Bench,) Senior Wrangler, 1787, Senior Smith's Prizeman; Lord Lyndhurst, (late Lord Chancellor,) 2nd Wrangler, 1794, Junior Smith's Prizeman; Sir John Beckett, (Judge Advocate,) 5th Wrangler, 1795; the late Sir John Williams, (Judge, Queen's Bench,) 18th Senior Optime, 1798; the late Sir N. C. Tindal, (Chief Justice, Common Pleas,) 8th Wrangler, 1799, Senior Medallist; the late Sir L. Shadwell, (Vice-Chancellor of England,) 7th Wrangler, 1800, Junior Medallist; Starkie, (Downing Professor of Law, University Counsel,) Senior Wrangler, 1803, Senior Smith's Prizeman; Lord Wensleydale, 5th Wrangler, 1803, Senior Medallist; the late Sir T. Coltman, (Judge, Common Pleas,) 13th Wrangler, 1803; Lord Chief Baron Pollock, Senior Wrangler, 1806, Senior Smith's Prizeman; Lord Langdale, Senior Wrangler, 1808, Senior Smith's Prizeman; the late Baron Alderson, Senior Wrangler, 1809, Senior Smith's Prizeman, and Senior Medallist; Sir W. H. Maule, (Judge, Common Pleas,) Senior Wrangler, 1810, Senior Smith's Prizeman; Baron Platt, (Exchequer,) 5th Junior Optime, 1810; Chambers, (Judge of Supreme Court, Bombay,) 5th Wrangler, 1811; Lord Cranworth, 17th Wrangler, 1812; Mirehouse, (Author of Law of Tithes, and Common Serjeant of City of London,) 13th Senior Optime, 1812; Sir J. Romilly, (Downing Professor of Law, and Professor of Law, University College, London,) 4th Wrangler, 1813; Vice-Chancellor Kindersley, 4th Wrangler, 1814; Sir R. H. Malkin, (Chief Justice of Prince of Wales's Island,) 3rd Wrangler, 1818; Lord Justice Turner, 9th Wrangler, 1819; the late R. C. Hildyard, (Queen's Counsel,) 12th Senior Optime, 1823; Mr. John Cowling, Q.C., M.P., (University Counsel, and Deputy High Steward,) Senior Wrangler, 1824, Senior Smith's Prizeman; Vice-Chancellor Wood, 24th Wrangler, 1824; Vice-Chancellor Parker, 7th Wrangler, 1825; Mr. Loftus T. Wigram, Q.C., (M.P. for University,) 8th Wrangler, 1825; Chief Justice Martin, (New Zealand,) 26th Wrangler, 1829, 3rd in 1st class Classics, and Junior Medallist.

Dublin.—1795, Sir T. Lefroy, (Chief Justice of Queen's Bench,) gold medal; 1800, Sir J. L. Foster, (Judge, Common Pleas, M.P. for University, 1807,) gold medal; 1802, P. C. Crampton, (Queen's Counsel, Judge, Queen's Bench,) gold medal; 1803, F. Blackburne, (Lord Chancellor of Ireland,) gold medal; 1811, R. H. Greene, (Baron of Exchequer,) gold medal; 1823, J. H. Monahan, (Chief Justice Common Pleas,) gold medal.

